# Full-Range Public Health Leadership, Part 2: Qualitative Analysis and Synthesis

**DOI:** 10.3389/fpubh.2015.00174

**Published:** 2015-07-08

**Authors:** Erik L. Carlton, James W. Holsinger, Martha C. Riddell, Heather Bush

**Affiliations:** ^1^Division of Health Systems Management and Policy, University of Memphis, Memphis, TN, USA; ^2^Department of Preventive Medicine, University of Kentucky, Lexington, KY, USA; ^3^Department of Health Management and Policy, University of Kentucky, Lexington, KY, USA; ^4^Department of Biostatistics, University of Kentucky, Lexington, KY, USA

**Keywords:** public health leadership, multifactor leadership questionnaire, public health workforce development, transformational leadership, local health department, full-range leadership

## Abstract

Public health leadership is an important topic in the era of U.S. health reform, population health innovation, and health system transformation. This study utilized the full-range leadership model in order to examine the public health leadership. We sought to understand local public health leadership from the perspective of local health department leaders and those who work with and for them. Public health leadership was explored through interviews and focus groups with directors (*n* = 4) and staff (*n* = 33) from local health departments. Qualitative analytic methods included reflexive journals, code-recode procedures, and member checking, with analysis facilitated by Atlas.ti v.6.0. Qualitative results supported and expanded upon previously reported quantitative findings. Leading by example and providing individual consideration to followers were found to be more important than other leader factors, such as intellectual stimulation, inspirational motivation, or idealized attributes of leaders. Having a clear and competent vision of public health, being able to work collaboratively with other community agencies, and addressing the current challenges to public health with creativity and innovation were also important findings. Idealized leadership behaviors and individual consideration should be the focus of student and professional development. Models that incorporate contextual considerations, such as the situational leadership model, could be utilized to ensure that optimal individual consideration is given to followers.

## Introduction

In an era of health reform, population health innovation, and health system transformation, issues, such as workforce development, management, and leadership, are central to the future of public health (1, 2). Public health problems are at once fascinating, frustrating, and inspiring, requiring public health leaders to engage multiple stakeholders in activities that, by their very nature, are public and open to broad scrutiny and debate (3). Effective public health leadership has never been more important, as current public health issues require leaders who are as skilled and astute politically as they are at managing the technical and logistical aspects of public health (4–6). Knowing the requisite skills, abilities, and styles of leaders can assist public health governance boards to select ideal candidates for leadership positions, aid public health leaders in promoting and developing leaders within their organizations, and guide trainers and educators in designing programs that ensure a workforce capable of leading public health in the future. Consequently, a better understanding of public health leadership is warranted to assure that leaders are able to meet current public health challenges.

Leadership is now at the forefront of research and practice-oriented efforts related to public health systems, services, and workforce issues (7–10). Previous studies have consistently drawn a link between a competent public health workforce and better public health outcomes, including improvements in the overall public health infrastructure (11–14).

The full-range leadership model establishes transformational, transactional, and passive-avoidant leadership styles, along with nine sub-styles of these categories, and examines the direct and indirect influence(s) of certain leadership styles on interpersonal relationships and organizational performance. [See Part 1 of this study, Carlton et al. (15), for a detailed description of each of these leadership styles and sub-styles]. Transformational leadership is often linked to improved quality, employee satisfaction, increased productivity, and better perceived leadership efficacy (16–21) while transactional and passive-avoidant (laissez-faire) leadership is seen as a prescription for mediocrity (22, 23). Academic competency models and public health workforce development programs frame a vision for public health leadership that preferences transformational, change-agent leaders who possess characteristics of transformational leadership, such as charisma and vision (24–26).

The amount of personal and/or positional power, the amount of employee resistance, the level and direction of employee motivation, the type of job, and even the personal and positional distance between leaders and followers can all significantly determine the type of leadership style that is not only feasible, but also preferable (27–30). Previous research has demonstrated the reciprocal influence of leaders and followers and each other’s behaviors and performance (31). Further, individual follower development, values, and personality, including connectedness and affect, may largely determine the extent to which transformational and/or transactional leadership is required (32–35). Similarity between leader and follower(s) in terms of values, personality, and goals is a strong predictor of preferred and actual leadership style (36). Further, a shared understanding of leadership style between leader and follower(s) is more strongly correlated with performance, behavior, commitment, and trust for transactional than for transformational leadership (37). Employee motivation, commitment, and relationships with supervisors are powerful determinants of transformational leadership (38). Consequently, leadership behavior may be heavily dependent on specific situations. Some authors even suggest that gender is an important consideration in the leader-follower dynamic (39).

Organizational and cultural influences on leadership, leadership expectations, leadership behaviors, and leadership efficacy must also be inserted into any equation of leadership theory. Studies have shown that cultural expectations of leadership can have a large impact on trust in leaders as well as perceptions of leader competence (40–42). The type of organization may be another important consideration (43). Some authors have found that a transformational style of leadership, as perceived by followers, is more prevalent in private than public health care settings (44). Indeed, organizational context may have a profound impact on perceived and actualized transactional and transformational leadership behaviors (40) and can greatly influence how, where, and what forms of leadership – either transactional or transformational – are possible (23, 45). Some authors have found that charismatic and transformational styles of leadership do not correlate with performance in public sector organizations (44, 46). One recent study found that in certain contexts, such as in the public sector, a transactional leadership style may be more predictive of organizational performance than a transformational leadership style (47). The posited ideal of transformational public health leadership may not fully account for how these follower and organizational dynamics influence the manner in which leadership can be actualized.

Despite this expansive body of literature on the full-range leadership model, no effort had yet been made to apply this model to the study of public health leadership. In the preceding companion paper, we reported the quantitative findings from a detailed mixed-method study on full-range leadership among local public health leaders. Specifically, transformational leadership styles among local health department leaders in one American state (Kentucky) were found to be highly correlated with better leadership outcomes (perceived performance, employee satisfaction, extra effort); whereas transactional leadership was not found to be correlated with these outcomes. These findings suggested that transformational leadership may be a more effective style among local health department leaders. Indeed, this is consistent with much of the literature, competencies, definitions, and guiding documents pertaining to public health leadership, which seem to preference transformational styles of leadership.

Still, while transformational leadership qualities may enable public health leaders to engage communities in efforts to improve population health, the full range of leadership qualities, including the technical and managerial acumen necessary not only to lead change, but to effectively attend to general and regular organizational tasks and responsibilities should not be overlooked. Indeed, Avolio and Bass (48) clearly state that leaders cannot be truly transformational without also possessing and leveraging strong transactional leadership abilities. Therefore, we hypothesized that given the often-technical demands of local public health delivery, a broader range of leadership abilities may be necessary, including additional transactional leadership qualities, which may often be overlooked in idealized visions of leaders. Consequently, an understanding of local public health leadership informed by public health leaders and their followers is warranted.

### Purpose

This study utilized the full-range leadership model in order to examine public health leadership. Our purpose was to understand local public health leadership styles from the perspectives of local health department leaders and their staffs. Guided by an interest in optimizing workforce development and leadership training activities, our primary research question was, “What leadership style(s) is/are most appropriate for effective leadership in local health departments?”

## Materials and Methods

Following the quantitative identification of transformational and transactional leadership tendencies in health department directors (see Part 1), interviews and focus groups were used to more fully understand the nature and practice of such leadership styles in public health settings. The purpose of these interviews and focus groups was to illuminate the situations and circumstances that facilitate or inhibit these styles from being employed. Using a semi-structured interview and discussion guide (Box 1), four directors were interviewed regarding their perspectives and experiences with public health leadership. Additionally, in each of these local health departments, focus groups were conducted with as many members of the executive team and general staff of the health departments as feasible, generally 10 to 12 individuals. A total of 33 individuals participated in four focus groups. The focus of these groups was also on perspectives and experiences with public health leadership, and discussion was likewise facilitated using the same semi-structured guide (Box 1).

Box 1 **Semi-structured interview/focus group discussion guide.**

1. Please describe the ideal qualities of public health leaders.
2. What is expected of leaders in your health department? How do you know this? [After reviewing definitions of transformational and transactional leadership styles]:
3. What are your thoughts on these leadership styles?
4. What type of leadership do you perceive to be generally employed in your health department?
5. What factors do or may encourage the use of a more transformational style of leadership in your health department?
6. What factors do or may encourage the use of a more transactional style of leadership in your health department?
7. How is leadership (or how are leaders) developed in your health department?

### Participants

Four local health department directors who participated in the quantitative phase of this study were randomly selected and interviewed. Upon completion of the interviews, the directors were asked if they would allow their employees to participate in a focus group concerning public health leadership. Dates and times were coordinated with the director and advertised to all employees via posters and email. Participation in the focus group activities was maximized by arranging to have the focus group convene at a mealtime in a conference room in the local health department and by coordinating work schedules and availability of interested employees with the health department director. No incentives were provided for either interview or focus group participants. To ensure that employees were open to discuss their thoughts and feelings, the directors did not attend the focus groups.

Demographics of interview and focus group participants were obtained including: gender (male or female), age (18–25, 26–35, 36–45, 46–55, and over 55), highest education completed (high school, associate’s degree, bachelor’s degree, master’s degree, doctoral degree, or their equivalents), whether participants had a professional public health degree (MPH, DrPH), and how many years of public health work experience they had (<1, 1–5, 6–10, 11–20, more than 20).

### Qualitative methods and analysis

The primary method of qualitative data collection was semi-structured interviews with local health department directors and focus groups with local health department staff. According to Creswell (49), interviews have the advantage of allowing researchers to gain historical information and to control the line of questioning. Conversely, interviews are more intimate and personal than surveys. Given that the amount of information the participant feels able or willing to share may be limited by the level of trust and/or credibility that the researcher has established with the participant(s), we used an approach that allowed interviews and focus groups to be more conversational and fluid. Leveraging an active, depth interviewing approach (50, 51), these interviews and focus groups followed a general outline of open-ended questions; then, as led by participants, the researcher more deeply explored the aspects of their experience and/or perspective not otherwise captured by a previously scripted question. Interviews and focus groups varied in length and detail depending on participant responses and the depth achieved in the interview. Most interviews lasted approximately 1 h, with focus groups averaging about an hour and a half.

In the emergent nature of qualitative research, data analysis is a highly reflective process for the researcher (49). To support this critical reflective process and to help ensure rigor, a reflexive field journal (52) was used to record the researcher’s reactions to each interview, the themes that seemed to be developing, and the researchers’ thoughts and reflections. These processes allowed the researchers not only to recall the aspects of each interview or focus groups, but also to identify potential biases that could have colored the research. Interviews were recorded on a digital audio recorder while the researchers simultaneously made hand-written field journal notes. Krefting (52) suggests that these notes aid researchers in recalling specific themes from the interviews/focus groups, to guide follow-up questions during the interviews/focus groups, and to aid in processing his/her experience of these interactions with participants.

Upon completion of the interviews and focus groups, the audio files were transcribed professionally and reviewed to ensure the accuracy, completeness, and timeliness of data analysis. Transcription quality is a fundamental component of qualitative rigor. Not only is accurately capturing what was said important, but also just as important is capturing how it was said (53). The digital audio files were electronically transmitted using secure file transfer protocols to a professional medical transcriptionist. To add additional layers of confidentiality, the digital files were devoid of audio or electronic identifiers that could be linked to the participant(s).

To facilitate the analysis and ensure the validity and credibility of the qualitative data gathered in this study, the Atlas.ti v6.0 software package was used to facilitate retrieval, filtering, and grouping of participants’ statements, thus enabling the development of qualitative themes and mapping key concepts. Researchers have noted the important role of computer-assisted qualitative data analysis software (CAQDAS) packages have afforded researchers to enhance the efficiency, depth, and rigor of their qualitative studies (54, 55).

To ensure the reliability of the data, a code–recode procedure was used. This procedure involved coding a portion of the interview data using an initial coding scheme developed based on our initial review of the transcripts and field journal notes from the interviews and focus groups. We then returned to the data 2 weeks later and recoded it. The results of these distinct coding sessions were compared to ensure that coding was performed consistently. A formal coding scheme was then finalized and the data analyzed using the software.

As core themes and concepts emerged from the data analysis, a member-checking procedure (52, 56) was used. This procedure involved providing data to research participants for comments and further response. Participant responses helped clarify themes and implications of results. Essentially, research participants were the ones who validated the study findings.

This study was conducted with approval of the University of Kentucky Institutional Review Board.

## Results

### Participant demographics

Demographic information of participants in this phase of the study is provided in Table 1. Thirty-seven local health department staff members participated in the qualitative phase of the study. This included four health department directors who had completed the earlier survey phase. These directors were interviewed separately from their staff members, who participated in focus groups.

**Table 1:** **Demographics of Phase 2 participants (*n* = 37)**.

Background information	Participants (*n* = 37)
Gender
Male	5 (13%)
Female	32 (87%)
Age
18–25	0 (0%)
26–35	13 (35%)
36–45	8 (22%)
46–55	12 (32%)
55+	4 (11%)
Highest education completed
High school/associate’s degree	17 (46%)
Bachelor’s degree	11 (30%)
Master’s degree	9 (24%)
Doctoral degree	0 (0%)
Public health degree (MPH, DRPH)
Yes	7 (19%)
No	30 (81%)
Years of public health work experience
<1	0 (0%)
1–5	12 (32%)
6–10	8 (22%)
11–20	15 (41%)
20+	2 (5%)

### Who are public health leaders?

Without fail, participants across all focus groups and interviews identified leadership less as a positional attribute and more as a personal quality. That is to say, an individual’s title as a director of a public health department, a divisional manager within a health department, or even a public health nurse without supervisory responsibilities does not determine whether or not that individual is a leader in the public health department. One nurse manager participating in a focus group said,

…Maybe even leadership doesn’t necessarily have to be in a supervisory role…I have a group of clinic nurses who all work doing the same job and yet there are some who have skills that are easily evident, identifiable, and they are willing to step up into leadership roles and that doesn’t mean take a supervisory position. Supervision is not necessarily leadership, leadership is not necessarily tied to titles.

This comment was echoed by other focus group participants. Given the focus group comments, leadership in local public health departments is as much a function of the personal qualities and behaviors of individuals as it is of the positions or titles they hold.

### Ideal qualities of public health leaders

Participants in interviews and focus groups identified several qualities or attributes of public health leaders they felt were ideal. Those qualities most often discussed by participants (i.e., frequency or how many discrete times the qualities were mentioned by participants) are shown in Table 2. These include aspects of leadership focused on other people, such as providing for or facilitating staff development/training; people-oriented relationships skills and individualized consideration and sensitivity; and delegation, empowerment, collaboration, and engagement. Leader behaviors exhibited through leading by example, modeling, and mentoring were also identified as ideal qualities. Creativity and innovation were very frequently discussed by participants, as was having vision and foresight. Leader competence in the form of basic management skills, understanding the fundamentals of public health practice and public health systems, and credibility were seen as not only ideal, but essential to public health leadership. Other ideal qualities of public health leaders included: being motivational, inspirational, and passionate; having and using good communication skills; being adaptable, flexible, and open to change; being decisive and having good decision-making skills; and being open to the influence of others. Other qualities mentioned by participants, but not shown in Table 2, include: being accountable and fostering accountability, professionalism, having a sense of humor, being humble, showing initiative, being fair, and possessing self-awareness and integrity.

**Table 2:** **Ideal qualities of public health leaders, by number of times (frequency) mentioned by participants**.

Leader attributes	Frequency
Staff development focused, training	25
Individual consideration, relationship skills, people-oriented, supportive, encouragement, sensitivity	25
Delegation, empowerment, engagement, collaboration	22
Creative and innovative	20
Leading by example, modeling, mentoring	18
Practical management skills, competence, basics of public health, knowledgeable, credibility, work ethic	17
Vision, foresight	15
Motivational, inspirational, passionate	15
Communication skills, incl. clarity, listening	13
Adaptability, flexibility, open to change	13
Decisiveness, good decision-making skills	9
Open to influence	7

Several themes concerning public health leadership styles and approaches emerged from the interviews and focus groups. Themes included balancing transformational and transactional leadership styles, leading by example, collaboratively engaging with followers, using transactional leadership when appropriate, and providing individual consideration to followers through situational-type leadership. Each of these themes is discussed in detail below.

### Balance of leadership styles

Much is expected of public health leaders. One public health director described his leadership role very succinctly, capturing several of the thoughts offered by other interview and focus group participants. Of his job as director, he said:

I’ve always perceived my job as being broken down into thirds. One-third is day-to-day management, where I answer emails, answer phone calls, take appointments, work with students and whatever other issue comes up. One-third of my job is doing what I call staff development: looking for places within the organization where I can acknowledge efforts of employees and just go in and visit with people and talk with people and sending our employee awards and doing things along that line. The other third is trying to figure out how to be better, how to be innovative, how to make the community better.

When given definitions of transformational and transactional leadership styles, most participants showed a preference for the transformational style of leadership. While the attributes and behaviors of transformational leaders are preferenced, nearly all participants indicated that in local public health departments, leaders need a more blended leadership style, one that could be adapted to situational requirements, including major public health events, individual staff needs, and the general activities of certain departments. It was noted that leaders need to be aware of their preferred style, so that they can develop and utilize other leadership styles as needed.

The need for a blended or versatile leadership style was also recognized by front-line staff members. Highlighting how the situation or context would dictate the appropriate leadership style, one participant stated: “It is very difficult, no matter how hard you try to be inspirational or motivational and challenge folks, in terms of doing the job every day. You reach a limit where you have to stop challenging people and motivating them and just say, ‘Just do it.”’

Participants were clear that transformational and transactional leadership styles are not mutually exclusive. Some specific circumstances may require different leadership styles. For example, one focus group participant explained how public health accreditation efforts might require both transformational and transactional leadership in order to be successful:

We’re on the road to accreditation and one of the things with that is standardization. Everybody doing the same thing so it’s more transactional, but the journey to get there and how to motivate each of those employees and supervisors to help us reach that step could definitely be transformational.

Finally, participants felt that leaders’ desired outcomes or goals may well be what ultimately determine the use of different leadership styles.

### Visionary leadership

Nearly all those who participated in this phase of the study identified visionary leadership as an ideal quality of leaders. Vision in public health leadership seems to be as much about the internal organization as it is about the larger organizational context – the community served by the health department. The motivation to innovate or be creative can come from this vision of the public’s health and a desire to have a positive influence on the population. It may also be an unwillingness to let the current health status continue:

A public health leader needs is to be dissatisfied with the state of affairs…If you’re satisfied it’s unlikely that you will ever be creative or motivated to try to be something better or different. If you’re just willing to be the *status quo*, the *status quo* is not good enough. You have to understand that. It’s not. The *status quo* is not good enough.

### Leading by example

One of the most consistent and necessary aspects of leadership addressed by participants was that of leading by example. As much as any other quality, participants felt that a leader’s behaviors were essential to their leadership. Said one director, “You have to get out and work side-by-side with your people. You have to demonstrate to them that you’re willing to do everything that you’re asking them to do…I know that I set the tone through my words and actions.”

Both directors and staff members addressed the importance of leading by example. Staff members especially indicated how the criticality of leading by example to team building and leader credibility. One supervisory staff member, who happens to work for the director quoted just above, said, “Leaders are expected to be role models. They’re expected to model the behavior that we’d like to see out of anyone.”

### Collaboration and engagement

One of the most important ways leaders are able to lead by example is by listening to, engaging, and collaborating with others. Participants said that this was as important for internal staff members as it was for external community partners. Building collaborative relationships with community partners was part of the visionary leadership quality described above. Participants indicated that a leader needs to have a broad vision of public health that encompasses both the public health agency and also includes the entire public health system. Participants also expressed the importance of being open to influence by others to allow for collaboration to occur.

### The role of transactional leadership

The context of public health work is an important consideration for public health leaders. Several participants spoke vociferously about how the daily realities of public health work necessitate various leadership styles. One participant, a clinical nurse manager, felt that the daily work of public health could be so codified that it might largely determine how leaders need to approach their roles. She said, “The transformational sounds wonderful if you had the time to sit and conceptualize and plan and think all day long, but a lot of work of the day is actually the transactional work just because things have to get done.” She strongly suggested that effective transactional leadership may be very important to much of public health practice. Indeed, the pervasiveness of transactional leadership in public health was evident in discussions with focus group participants. A number of front-line staff with some supervisory experience, most with over 10 years of public health experience, did not exclude transformational leadership, but they suggested that many tasks in public health practice did not lend themselves to being transformed due to the daily realities of public health practice.

### Individual consideration and the situational leader

One supervisory staff member suggested that the approach to each person must be unique, highlighting one of the overarching themes of the qualitative phase of this study. Each situation is different and thus may demand a very different leadership approach, particularly when working with others. The idea of individual consideration, one of the constructs of transformational leadership, was at the forefront of participants’ minds. An important part of individual consideration is, as one participant said, “Finding strengths in everyone and working to maximize those strengths so that everybody is contributing.” Consequently, leaders who are able to consider individual circumstances and adapt their leadership accordingly, may be most effective in their leadership roles.

### Summary of key findings

In summary, several key findings emerged from the qualitative phase of this study. Perhaps, most importantly, participants clearly indicated that local public health leaders should balance transformational and transactional leadership styles. Transformational styles are best received when leaders lead by example and when they collaboratively engage their followers. However, there are times when transactional leadership may be necessary to assure performance or to accomplish specific tasks. According to participants, what is critical is the leader’s ability to provide each follower individual consideration and approach that these individual leadership experiences situationally.

## Discussion

Using a two-phase, sequential, mixed-method design, this study examined public health leadership through the perspective of local health department directors and their staffs and through lens of the full-range leadership model (48, 57, 58). While this study found a variety of leadership styles and outcomes of leadership among local health department directors, specific aspects of transformational leadership, namely idealized leader behaviors and individual consideration, were significant constructs emerging from the study.

The public health directors interviewed consistently considered leading by example as one of the most important aspects of leadership in public health. Public health practitioners often face many challenges in their work environment, including lower wages than private sector peers, antiquated facilities, prescribed operational protocols, and a severely disadvantaged client population. Followers were clear that when leaders are not willing to do what they ask others to do, they destroy trust, which erodes and may ultimately undermine the leaders’ influence. These concepts mirror the models of leader integrity proposed by Grover and Moorman (59) and others (60).

Leaders and followers alike highlighted the role of leader self-awareness and individual consideration. Participants suggested that for leaders to be most effective, they needed to know and operate from their own style preference, as much as is appropriate, while working to determine and utilize the approach(es) that best fit each of their individual followers. This sort of resonance can allow leaders to transcend “siloed” agency functions and create unifying organizational cultures where individual strengths and skills are synergized instead of cannibalized. The concept of authentic leadership is evident in these findings. Authenticity, and thus resonance, in leadership derives from having a clear awareness of strengths and growth areas, values, vision, and expectations of self and others. The presence of these factors can have a profound impact on followers and is deeply empowering to leaders (61).

As leadership was discussed with participants, it became evident that as important as leader behaviors were to leadership outcomes, such as employee trust, satisfaction, and engagement, the contexts in which leader-follower interactions took place were just as important. Many participants discussed the role of relationship building and so-called “people-skills” in determining how leaders should work with others. Also central to the equation were various circumstances inherent and unique to public health practice, such as broad variance in work-related tasks (i.e., sanitation and inspection compared to public health nursing), outbreaks or other public health emergencies, or the demands of community-based collaborative health education and promotion efforts. These vastly differing situations likely necessitate a more adaptive, flexible, and balanced leadership approach that embodies the best of both transformational and transactional leadership, suggesting that a situational leadership approach can be very effective in public health settings.

### Implications for practice

This study has important implications for public health practitioners. One of the most significant implications of the study is related to workforce development. Study findings clearly indicate the aspects of leadership that need to be the focus of student and professional workforce development activities, namely idealized leadership behaviors (e.g., leading by example, developing a strong sense of purpose and mission, and making sound decisions) and individualized consideration (e.g., paying attention to each individual’s need for achievement and growth by identifying strengths and through mentoring and staff development). In terms of leadership and organizational outcomes, these two aspects of leadership rose above all other aspects of leadership examined. That participants highlighted the parallel importance of more transactional leadership styles suggests that leadership development models should be mindful to be inclusive of these concepts and not merely preference the transformational leadership qualities that have come to dominate public health leadership development approaches.

#### Continuing Education

Investments in staff development are more comprehensive than leadership or relationship skills education. Participants’ responses indicated that simply providing opportunities for staff members to develop skills and abilities not only enhances the level of technical expertise available to improve public health practice, but also enhances loyalty to and engagement with the agency. Employees whose strengths are identified and developed feel greater self-efficacy and, consequently, more empowerment to contribute to the mission of the organization. Therefore, opportunities for specialized training or advanced education, even if the organization is unable to fund these activities, may be useful.

#### Selection and Hiring

This study has important implications for state health officials and for local boards of health, which may be involved in the selection, and development of new local public health directors. Many members of local health boards are not trained in public health and may be appointed to fill codified positions on the local board; yet, they are tasked with hiring and supervising local directors, which may have a significant positive or negative impact on the public health agency.

### Implications for future research

Missing from the literature are accurate measures of return on investment for public health workforce development activities. It is one thing to enumerate leadership styles in the hope of developing leaders who more closely portray a given model of leadership. It is entirely different to tie these findings to consistent and objective measures that are meaningful in the management of public health practice. As funding becomes increasingly limited and risks being diverted from leadership development activities, measures should be developed to objectively evaluate the impact of leadership development activities. Studies could look to examine the role of leadership related to improvements in organizational financial performance, staff turnover, employee satisfaction, or other common and consistent metrics. Research into these topics should contribute substantively to the pillars of workforce research and development: enumeration, competency, and capacity.

This study’s finding that a more blended, situational style of leadership may be necessary for effective local public health practice draws attention to the role of translational and interdisciplinary research. While the full-range model of leadership that undergirded this study has been used effectively in business and educational research and development activities, it had yet to be employed in a public health setting. Models from business administration, educational leadership, and other fields are constantly being developed, applied, refined, and disseminated. Consequently, business leadership theories and practices may be evolving much more rapidly than similar theories in the health and helping professions, especially public health. This suggests that public health workforce researchers should find ways to translate cutting-edge theories from other disciplines, particularly business administration and educational leadership, so that public health leadership and workforce development efforts keep pace with and inform the larger leadership literature.

### Limitations

Limitations include the limited number of focus groups (*n* = 4) conducted. Though relatively robust, the final qualitative sample size (*n* = 37) was somewhat smaller than anticipated. A larger sample of focus groups conducted in a larger number of locations should enhance the qualitative contribution of the study. The study may also be limited in that only suburban and rural health departments were represented, with no representation from urban health departments. Urban environments may demand a different form of leadership – either more transformational or more transactional – than health departments from other geographic locations.

## Conclusion

The demands on public health leadership are evolving as rapidly as the environment of health reform, population health innovation, and health system transformation in which it operates. As we develop current and future public health leaders, we would be wise to incorporate a holistic approach to leadership development and to focus on factors that seem to stand out to those working in these settings. For local public health practitioners, idealized leadership behaviors and individualized consideration should be the focus of workforce education and development efforts. Models that incorporate contextual considerations, such as the situational leadership model, should be utilized. Above all, in an era of transformational change in public health, we must remember the vital role of leadership for the public’s health and seek to develop leaders who possess the best of both transactional and transformational leadership styles.

## Conflict of Interest Statement

The authors declare that the research was conducted in the absence of any commercial or financial relationships that could be construed as a potential conflict of interest. The Review Editor William A. Mase declares that, despite having collaborated with the author James W. Holsinger, Jr., the review process was handled objectively and no conflict of interest exists.

## References

[1] CDC. Ten Essential Public Health Services. (2008). Available from: http://www.cdc.gov/od/ocphp/nphpsp/EssentialPHServices.htm

[2] IOM. The Future of the Public’s Health in the 21st Century. (2002). Available from: http://www.iom.edu/Object.File/Master/4/165/AssuringFINAL.pdf

[3] Koh HK Jacobson M Fostering public health leadership. J Public Health (2009) 31(2):199–201. 10.1093/pubmed/fdp032 19451343

[4] Annett H Leadership in public health: a view from a large English PCT co-terminous with a local authority. J Public Health (2009) 31(2):205–7. 10.1093/pubmed/fdp033 19414480

[5] Hunter DJ Leading for health and wellbeing: the need for a new paradigm. J Public Health (2009) 31(2):202–4. 10.1093/pubmed/fdp036 19372148

[6] Williams JC Costich J Hacker WD Davis JS. Lessons learned in systems thinking approach for evaluation planning. J Public Health Manag Pract (2010) 16(2):151–5. 10.1097/PHH.0b013e3181c6b50d 20150798

[7] Council on Linkages Between Academia and Public Health Practice. Core Competencies for Public Health Professionals. (2010). Available from: http://trainingfinder.org/competencies/list.htm

[8] Kristine MG Bernard JT. The public health workforce, 2006: new challenges. Health Aff (2006) 25(4):923. 10.1377/hlthaff.25.4.923 16835170

[9] Lichtveld MY Cioffi JP Baker EL Jr Bailey SB Gebbie K Henderson JV Partnership for front-line success: a call for a national action agenda on workforce development. J Public Health Manag Pract (2001) 7(4):7. 10.1097/00124784-200107040-00002 11434035

[10] Mays G Smith S Ingram R Racster L Lamberth C Lovley E. Public health delivery systems: evidence, uncertainty, and emerging research needs. Am J Prev Med (2009) 36(3):256–65. 10.1016/j.amepre.2008.11.008 19215851

[11] Baker ELJ Stevens RHE Linking agency accreditation to workforce credentialing: a few steps along a difficult path. J Public Health Manag Pract (2007) 13(4):430–1. 10.1097/01.PHH.0000278040.84636.23 17563635

[12] Baker EL Jr Potter MA Jones DL Mercer SL Cioffi JP Green LW The public health infrastructure and our nation’s health. Annu Rev Public Health (2005) 26(1):303–18. 10.1146/annurev.publhealth.26.021304.144647 15760291

[13] Cioffi JP Lichtveld MY Tilson H A research agenda for public health workforce development. J Public Health Manag Pract (2004) 10(3):186–92. 10.1097/00124784-200405000-00002 15253514

[14] Scutchfield FD Bhandari MW Lawhorn NA Lamberth CD Ingram RC. Public health performance. Am J Prev Med (2009) 36(3):266–72. 10.1016/j.amepre.2008.11.007 19215852

[15] Carlton EL Holsinger J Riddell M Bush H. Full-range public health leadership, Part 1: quantitative analysis. Front Public Health (2015) 3:73. 10.3389/fpubh.2015.00073 25984511 PMC4415305

[16] Bass BM Jung DI Avolio BJ Berson Y. Predicting unit performance by assessing transformational and transactional leadership. J Appl Psychol (2003) 88(2):207–18. 10.1037/0021-9010.88.2.207 12731705

[17] Barbuto JE Jr Motivation and transactional, charismatic, and transformational leadership: a test of antecedents. J Leader Organ Stud (2005) 11(4):26–40. 10.1177/107179190501100403

[18] Boerner S Eisenbeiss SA Griesser D Follower behavior and organizational performance: the impact of transformational leaders. J Leader Organ Stud (2007) 13(3):15–26. 10.1177/10717919070130030201

[19] Gellis ZD Social work perceptions of transformational and transactional leadership in health care. Soc Work Res (2001) 25(1):17 10.1093/swr/25.1.17

[20] Lowe KB Galen Kroeck K Effectiveness correlates of transformational and transactional leadership: a meta-analytic. Leadersh Q (1996) 7(3):385 10.1016/S1048-9843(96)90027-2

[21] Mary NL Transformational leadership in Human Service Organizations. Adm Soc Work (2005) 29(2):105–18. 10.1300/J147v29n02_07

[22] Bass BM From transactional to transformational leadership: learning to share the vision. Organ Dyn (1990) 18(3):19–31. 10.1016/0090-2616(90)90061-S

[23] Friedman AA Beyond mediocrity: transformational leadership within a transactional framework. Int J Leader Educ (2004) 7(3):203–24. 10.1080/1360312042000213877

[24] ASPPH. MPH Core Competency Model. (2006). Available from: http://www.aspph.org/wp-content/uploads/2014/04/Version2.31_FINAL.pdf

[25] ASPPH. DrPH Core Competency Model. (2009). Available from: http://www.aspph.org/wp-content/uploads/2014/04/DrPHVersion1-3.pdf

[26] Tilson H Gebbie KM The public health workforce. Annu Rev Public Health (2004) 25(1):341–56. 10.1146/annurev.publhealth.25.102802.124357 15015924

[27] Barbuto JE Jr Fritz SM Matkin GS. Leaders’ bases of social power and anticipation of targets’ resistance as predictors of transactional and transformational leadership. Psychol Rep (2001) 89(3):663. 10.2466/pr0.2001.89.3.663 11824734

[28] Howell JM Hall-Merenda KE The ties that bind: the impact of leader-member exchange, transformational and transactional leadership, and distance on predicting follower performance. J Appl Psychol (1999) 84(5):680–94. 10.1037/0021-9010.84.5.680

[29] Shivers-Blackwell SL Using role theory to examine determinants of transformational and transactional leader behavior. J Leader Organ Stud (2004) 10(3):41–50. 10.1177/107179190401000304

[30] Wylie DA Gallagher HL Transformational leadership behaviors in allied health professions. J Allied Health (2009) 38(2):65–73. 19623787

[31] Conger JA Kanungo RN Menon ST Charismatic leadership and follower effects. J Organ Behav (2000) 21(7):747–67. 10.1002/1099-1379(200011)21:7<747::AID-JOB46>3.0.CO;2-J

[32] Dvir T Shamir B Follower developmental characteristics as predicting transformational leadership: a longitudinal field study. Leadersh Q (2003) 14(3):327–44. 10.1016/S1048-9843(03)00018-3

[33] Ehrhart MG Klein KJ Predicting followers’ preferences for charismatic leadership: the influence of follower values and personality. Leadersh Q (2001) 12(2):153–79. 10.1016/S1048-9843(01)00074-1

[34] Epitropaki O Martin R The moderating role of individual differences in the relation between transformational/transactional leadership perceptions and organizational identification. Leadersh Q (2005) 16(4):569–89. 10.1016/j.leaqua.2005.06.005

[35] Piliai R Schriesheim CA Williams ES Fairness perceptions and trust as mediators for transformational and transactional leadership: a two-sample study. J Manage (1999) 25(6):897 10.1177/014920639902500606

[36] Waismel-Manor R Tziner A Berger E Dikstein E Two of a kind? Leader–member exchange and organizational citizenship behaviors: the moderating role of leader–member similarity. J Appl Soc Psychol (2010) 40(1):167–81. 10.1111/j.1559-1816.2009.00568.x

[37] Whittington JL Coker RH Goodwin VL Ickes W Murray B Transactional leadership revisited: self–other agreement and its consequences. J Appl Soc Psychol (2009) 39(8):1860–86. 10.1111/j.1559-1816.2009.00507.x

[38] Piccolo RF Colquitt JA Transformational leadership and job behaviors: the mediating role of core job characteristics. Acad Manage J (2006) 49(2):327–40. 10.5465/AMJ.2006.20786079

[39] Eagly AH Johannesen-Schmidt MC van Engen ML. Transformational, transactional, and laissez-faire leadership styles: a meta-analysis comparing women and men. Psychol Bull (2003) 129(4):569. 10.1037/0033-2909.129.4.569 12848221

[40] Atwater LE Wright WJ Power and transformational and transactional leadership in public and private organizations. Int J Publ Admin (1996) 19(6):963–89. 10.1080/01900699608525127

[41] Casimir G Waldman DA Bartran T Yang S Trust and the relationship between leadership and follower performance: opening the black box in Australia and China. J Leader Organ Stud (2006) 12(3):68–84. 10.1177/107179190601200305

[42] Yunxia Z Do cultural values shape employee receptivity to leadership styles? Acad Manag Perspect (2007) 21(3):89–90. 10.5465/AMP.2007.26421244

[43] Corrigan PW Diwan S Campion J Rashid F. Transformational leadership and the mental health team. Adm Policy Ment Health (2002) 30(2):97–108. 10.1023/A:1022569617123 12680615

[44] Al-Mailam FF. Transactional versus transformational style of leadership – employee perception of leadership efficacy in public and private hospitals in Kuwait. Qual Manag Health Care (2004) 13(4):278–84. 10.1097/00019514-200410000-00009 15532520

[45] Johns C In search of transformational leadership in a transactional world. The International Association for Human Caring Annual Meeting: the power of caring: gateway to healing, May 16 to May 19, 2007, Millenium Hotel, St. Louis, MO, USA. Int J Hum Car (2007) 11(3):59–59.

[46] Javidan M Waldman DA Exploring charismatic leadership in the public sector: measurement and consequences. Public Adm Rev (2003) 63(2):229–42. 10.1111/1540-6210.00282

[47] Vecchio RP Justin JE Pearce CL The utility of transactional and transformational leadership for predicting performance and satisfaction within a path-goal theory framework. J Occup Organ Psychol (2008) 81(1):71–82. 10.1348/096317907X202482

[48] Avolio BJ Bass BM Multifactor Leadership Questionnaire: Manual & Sample Set. 3rd ed Menlo Park, CA: Mind Garden Inc (2004).

[49] Creswell JW Research Design: Qualitative, Quantitative, and Mixed Methods Approaches. 2nd ed Thousand Oaks, CA: SAGE (2003).

[50] Holstein JA Gubrium JF Animating interview narratives. In: Silverman D, editor. Qualitative Research, 3rd edition. Thousand Oaks, CA: SAGE (2011). p. 149–67.

[51] Patton MQ How to Use Qualitative Methods in Evaluation. London: SAGE (1987).

[52] Krefting L. Rigor in qualitative research: the assessment of trustworthiness. Am J Occup Ther (1991) 45(3):214–22. 10.5014/ajot.45.3.214 2031523

[53] Poland BD Transcription quality. In: Gubrium JF Holstein JA, editors. Inside Interviewing: New Lenses, New Concerns. Thousand Oaks, CA: SAGE (2003). p. 267–87.

[54] Seale CF Computer-assisted anlaysis of qualitative interview data. In: Gubrium JF Holstein JA, editors. Handbook of Interview Research: Context and Method. Thousand Oaks, CA: SAGE (2002). p. 651–70.

[55] Weitzman EA Software and qualitative research. 2nd ed In: Denzin NK Lincoln YS, editors. Handbook of Qualitative Research. Thousand Oaks, CA: SAGE (2000). p. 803–20.

[56] Ellis C Bochner AP Autoethnography, personal narrative, reflexivity: researcher as subject. 2nd ed In: Denzin NK Lincoln YS, editors. Handbook of Qualitative Research. Thousand Oaks, CA: SAGE (2000). p. 733–68.

[57] Bass BM Bass R The Bass Handbook of Leadership. 4th ed New York, NY: Free Press (2008).

[58] Sosik JJ Jung D Full Range Leadership Development: Pathways for People, Profit and Planet. New York, NY: Psychology Press (2009).

[59] Grover SL Moorman R Challenges to leader integrity: how leaders deal with dilemmas of honesty. In: Garsten C Hernes T, editors. Ethical Dilemmas in Management. New York, NY: Routledge (2009). p. 52–63.

[60] Kouzes JM Posner BZ The Leadership Challenge. 4th ed San Francisco, CA: Jossey-Bass (2007).

[61] Palanski ME Yammarino FJ Integrity and leadership: clearing the conceptual confusion. Eur Manage J (2007) 25:171–84. 10.1016/j.emj.2007.04.006

